# Xiao Yao San Improves Depressive-Like Behaviors in Rats with Chronic Immobilization Stress through Modulation of Locus Coeruleus-Norepinephrine System

**DOI:** 10.1155/2014/605914

**Published:** 2014-12-25

**Authors:** Xiu-Fang Ding, Xiao-Hua Zhao, Yang Tao, Wei-Chao Zhong, Qin Fan, Jian-Xin Diao, Yuan-Liang Liu, Yu-Yao Chen, Jia-Xu Chen, Zhi-Ping Lv

**Affiliations:** ^1^School of Traditional Chinese Medicine, Southern Medical University, Guangzhou, Guangdong 510515, China; ^2^School of Preclinical Medicine, Beijing University of Chinese Medicine, Beijing 100029, China; ^3^Comprehensive Department of Traditional Chinese Medicine of Shantou City Hospital, Shantou 515000, China

## Abstract

Most research focuses on the hypothalamic-pituitary-adrenal (HPA) axis, hypothalamus-pituitary-thyroid (HPT) axis, and hypothalamus-pituitary-gonadal (HPGA) axis systems of abnormalities of emotions and behaviors induced by stress, while no studies of Chinese herbal medicine such as Xiao Yao San (XYS) on the mechanisms of locus coeruleus-norepinephrine (LC-NE) system have been reported. Therefore, experiments were carried out to observe mechanism of LC-NE system in response to chronic immobilization stress (CIS) and explore the antidepressant effect of XYS. Rat model was established by CIS. LC morphology in rat was conducted. The serum norepinephrine (NE) concentrations and NE biosynthesis such as tyrosine hydroxylase (TH), dopamine-*β*-hydroxylase (DBH), and corticotrophin-releasing-factor (CRF) in LC were determined. Results showed that there were no discernible alterations in LC in rats. The serum NE concentrations, positive neurons, mean optical density (MOD), and protein levels of TH, DBH, and CRF in model group were significantly increased compared to the control group. But XYS-treated group displayed a significantly decreased in NE levels and expressions of TH, DBH, and CRF compared to the model group. In conclusion, CIS can activate LC-NE system to release NE and then result in a significant decrease in rats. XYS treatment can effectively improve depressive-like behaviors in rats through inhibition of LC-NE neurons activity.

## 1. Introduction

Stress response is a risk factor that can develop affective or mental disorders, including anxiety, posttraumatic stress disorder, depression, and other disorders [1] and is characterized by the activation of the Locus coeruleus-norepinephrine (LC-NE) system. Indeed, LC-NE system can activate by multiple stressors, including social stress, footshock, novelty stress, and restraint. Furthermore, the effects of stress exposure can mimic the behavioral effects after activation LC, and damaged LC then attenuate neuroendocrine and behavioral responses [2, 3]. The locus coeruleus in brainstem noradrenergic neurons during stress period can supply norepinephrine (NE) across the central nervous system (CNS) to form a LC-NE system and modulate the central stress response.

The NE contains axons that are widely distributed, indicating that this neurotransmitter plays a pivotal role in CNS function and behaviors [4, 5]. The changes of behavioral depression found to be most closely related to stress were NE in the locus coeruleus. Norepinephrine-synthesizing enzymes including TH and DBH may be a neurochemical mechanism to interpret behavioral depression [6]. Both TH and DBH gene expressions in LC can be induced rapidly after immobilization stress [6, 7]. Additionally, the role of activation of central CRF systems associated with mediation of behavioral responses to stressors. Summarized evidences show that stress can activate two forms of CRF-norepinephrine interactions. Either autonomic or emotional stress activates CRF release in the LC region, which in turn stimulates or activates LC-NE projection system [8, 9]. Administration of CRF antagonists within the LC can attenuate increased LC-NE neurotransmission induced by stressor [10, 11].

Many of studies have demonstrated that repeated or prolonged stressor can lead to complicated alterations in LC-NE neurotransmission. Although repeated or chronic stressors may not elicit NE to release, they can increase the capacity of this system to release NE, due to increased rates of NE synthesis [4]. At present, most research focuses on the hypothalamic-pituitary-adrenal (HPA) axis [12], hypothalamus-pituitary-thyroid (HPT) axis, and hypothalamus-pituitary-gonad (HPGA) axis systems of abnormalities of emotions and behaviors induced by stress [13, 14], while no studies of Chinese herbal medicine such as XYS or compounds on the mechanisms of LC-NE systems have been reported.

XYS is a Chinese herbal formula that was initially recorded in the book* Taiping Huiming Heji Jufang* in the Song Dynasty (960–1127 A.D.) in ancient China. The XYS finished products including decoction, powder, and pill have been widely used to treat mental disorders for thousands of years in China [15–17]. The therapeutic effects of XYS on ameliorating depressive-like behaviors or regulating expression of biochemical marker under stress period had been proved. For example, XYS can effectively regulate expression of tyrosine hydroxyls (TrkB), neurotrophin 3 (NT-3), neurotrophic factor (BDNF), leptin receptor (ob-R), and neuropeptide Y (NPY) in the hippocampal, arcuate nucleus and frontal cortex in rats with CIS [18, 19]. XYS can inhibit IL-1*β* production in hippocampus and paraventricular nucleus (PVN) induced by repeated stress and alter metabolic network abnormalities in rats with chronic unpredictable mild stress (CUMS) or CIS [20–22]. In these investigations, the antidepressant effects of XYS on affective disorders were nearly all focused on hippocampus [21, 23], hypothalamus [24] (including arcuate nucleus [19]), and so on. While the research on Xiao Yao San regulates psychiatric affective disorders in mice locus coeruleus as an antidepressant medicine is lacking.

Towards that end, this study was designed to determine whether XYS can ameliorate depressive-like behaviors in rats with CIS through modulation of locus coeruleus-norepinephrine system based on our previous work [25].

## 2. Materials and Methods

### 2.1. Animals and Stress Procedure

The healthy Sprague Dawley male rats with bodyweight of 180–200 g were purchased from the Animal Center of Southern Medical University (number 2006-0015). All animals were fed in standard animal room (room temperature: 21 ± 1°C; relative humidity: 30%–40%; light condition: a 12 h light-dark cycle). Food and purified water were available* ad libitum*. This experiment was approved by the Animal Ethics Committee of Southern Medical University and was performed strictly and conformed to the guidelines for the Care and Use of Laboratory Animals of China. All rats were housed individually and habituated for one week before performing further experiment. A total of 48 rats were arbitrarily assigned into four groups according to their weight. These comprised control group, model group, XYS treatment group, and fluoxetine treatment group. The treated groups were also subjected to CIS. The rats in the control group were fed routinely for twenty-one days; others were restrained by forcing them into an immobilizer device. In order to avoid the restraint adaptability of rats, CIS protocol made a minor modification according to previous studies [21, 26]. In this experiment, immobilization time was randomly arranged and gradually increased on day 1st for 1 h to 6 h on day 21st. Rats in control and model group were given 2 mL physiological saline by intragastric administration. Rats in XYS treatment group were given XYS decoction with the same ways according to their body surface area (dosage: 10 g/kg/d). Rats in fluoxetine treatment group were given fluoxetine (5 mg/kg/d). The physiological saline and drugs were intragastrically administered at 4:00 pm daily.

### 2.2. Preparation of Xiao Yao San

The herbal formula of XYS comprises the following drugs: Angelicae sinensis Radix (root of* Angelica sinensis *(Oliv.) Diels), Paeoniae Radix Alba (root of* Paeonia lactiflora* Pall.), Bupleuri Radix (root of* Bupleurum* chinese DC), Atractylodis Macrocephalae Rhizoma (root and rhizome of* Atractylodes macrocephala *Koidz), Glycyrrhizae Radix et Rhizoma (root and rhizome of* Glycyrrhiza uralensis *Fish), Poria (fungus nucleus of* Poria cocos* (Schw.) Wolf), Zingiberis Rhizoma Recens (root and rhizome of* Zingiber officinale* Rosc.), and Menthae Haplocalycis Herba (overground parts of* Mentha haplocalyx* Briq.) (the ratio is 6 : 6 : 6 : 6 : 6 : 2 : 2 : 3). These herbal medicines were obtained from the Affiliated Nan Fang Hospital of Southern Medical University (Guangzhou, China) and authenticated by Liu Q, Department of Pharmacognostical Identification in School of Chinese Medicine of Southern Medical University. All herbal medicines were immersed in 10 times volume of distilled water and boiled at 80°C for 1 h; the water extracts were then collected. This process was repeated again [21]. The extracts were concentrated by rotary Evaporator, and the final concentration was 1 g/mL. Fluoxetine (Lilly Suzhou Pharmaceutical Co., LTD, number J20080016) was obtained from the Affiliated Nan Fang Hospital of Southern Medical University and dissolved in distilled water. The final concentration was 0.2 mg/mL. These solutions were stored at −4°C, and quality of XYS had been proved by high performance liquid chromatography-mass spectrometry analysis as reported in our previous research [23].

### 2.3. Depressive-Like Behaviors and Body Weight in Rat with CIS

Depressive-like behaviors in rat including action, diet, body weight, and defecation were observed during 21 d CIS period. The body weights of rats were measured on day 1st, day 7th, day 14th, and day 21st. To examine the depressive behaviors in rats, a sucrose preference test (SPT) was measured on days 7th, 14th, and 21st as previous described and made a minor modification [27]. Briefly, rats were singly habituated for 1 d prior to the test; their water bottles were replaced with two 50 mL bottles (one marked A: 1% sucrose solution; one marked B: water). The bottle positions were changed daily to prevent possible side bias. Rats were caged for 24 h to a 1% sucrose solution before measurement. This sucrose preference test was conducted weekly following water and food deprivation for 24 h. The total fluid intake in two bottles was measured as VolA and VolB. The sucrose preference for each rat was calculated as 100 × (VolA/(VolA + VolB)).

### 2.4. Noradrenaline (NE) Measurement

The experiment was terminated on the 21st day, and rats were anaesthetized with an intraperitoneal injection 10% chloral hydrate (0.35 to 0.40 mL/100 g bodyweight). Venous blood was drawn from abdominal aorta and serum was then separated to measure NE levels. Enzyme-linked immunosorbent assay (ELISA) (CUSABIO, Wu Han, China) was measured using a microtiter plate reader (Victor3V, Perkin Elmer, Waltham, MA, USA) according to the protocol in the kit.

### 2.5. H&E and Immunohistochemical Staining

Six animals in each group were anaesthetized with 2% pentobarbital sodium (40 mg/kg) and perfused with cold 0.9% NaCl solution followed by perfusate solution (4% paraformaldehyde, 2.5% glutaraldehyde, and 0.1 mol PBS). Brain stem contained locus coeruleus was cut out from brain tissues and fixed in 2.5% paraformaldehyde solution for 48 hours and was then cut into serial sections (5 *μ*m). Preparation of tissue section and procedure of H&E staining were described in previous report [28].

For immunohistochemistry, the paraffin-embedded locus coeruleus sections were processed as free-floating slices, including deparaffinized, rehydrated, antigen retrieval, and then treated with hydrogen peroxidase in DDW (double distilled water) for 10 min at room temperature in order to inhibit endogenous peroxidase. Antigen retrieval was conducted by heating for 15 min. Slices were incubated with primary antibodies (anti-TH, 1 : 1000 dilution; anti-DBH, 1 : 1000 dilution; anti-CRF, 1 : 500 dilution) after blocking in the antisera. After incubation with the secondary antibody, sections were placed in DAB reagent (ZSGB-BIO, Beijing, China) for 5–10 min at room temperature. After a further rinsing in PBS, sections were restained with hematoxylin and were mounted on gelatin-coated slides for observation under a light microscope.

### 2.6. Western Blot Analysis

Protein levels of DBH, CRF, and TH were measured by Western blotting. The procedure was performed as previously described [23]. Briefly, proteins were extracted from locus coeruleus tissues, and concentrations were detected using a BCA protein assay kit (Beyotime, Shanghai, China). Based on the molecular weight of target protein, 12% separating gels were prepared, and concentration of stacking gels was 5%. Protein gels were transferred to PVDF (polyvinylidene fluoride) membranes using a semidry blotter. For detection of the target protein, PVDF membranes were incubated in TBST with 5% skimmed milk for 1 h at room temperature. After washing seven times for 5 min each in TBST, membranes were incubated first with primary antibodies for 2 h at room temperature or at 4°C overnight. Primary antibodies were anti-TH (1 : 2000 dilution), anti-DBH (1 : 2000 dilution), anti-CRF (1 : 500 dilution), anti-beta-actin (1 : 500 dilution), respectively.

After washing three times for 5 min each in TBST, membranes were incubated with horseradish peroxidase- (HRP-) conjugated secondary antibody which was diluted in enclosed liquid (1 : 3000, 1 : 5000) for 1 h at room temperature. After three 5 min washes in TBST, membranes were developed using the enhanced chemiluminescence (ECL) detection reagent for 3 min and exposed to a CCD system (Image station 2000 MM, Kodak, Rochester, Rochester, NY, USA). The intensity of protein band was measured using an Image J software.

### 2.7. Statistical Analysis

All data were presented as the mean ± SD and analyzed using an SPSS 17.0 statistical package. The multicomparison was performed. The mean values were conducted using one-way ANOVA as well as the homogenous variances test. The nonhomogeneous variances were compared by using Welch's test. *P* < 0.05 was considered statistically significant difference.

## 3. Results 

### 3.1. Behaviors and Body Weight in Rat with CIS

**Figure 1:**
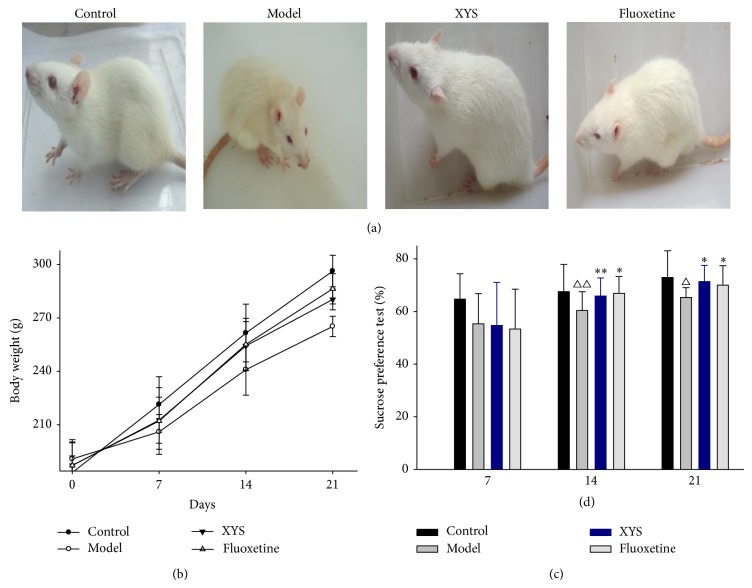
Effect of XYS on depressive-like behaviors of rats with CIS. (a) Actions and furs were observed in rats with CIS. (b) Rat body weight was recorded once a week during 21 d-CIS period. (c) Sucrose preference test was conducted once a week during 21 d-CIS period. Data were expressed as mean ± SD, *n* = 12 per group. ^△△^ *P* < 0.01, ^△^ *P* < 0.05 versus control, ^**^ *P* < 0.01, ^*^ *P* < 0.05 versus model.

Rats were active and were in normal mood conditions with lustrous furs before modeling. In order to know the general status in rats under immobilization stress period, the variations of general behaviors in rats before and after modeling were observed. Rats in four groups were in an agitated state and prone to irritate and struggle during 7 d CIS period. After 10 d CIS, rats in model group showed passive manifestations and negative mood conditions. Rats in model group presented a fatigue, weak sounds, sluggish response, poor appetite, loose stool, and dull and yellow furs after stress for 14 days (Figure 1(a)). Body weights were significantly decreased on days 14th and 21st after immobilization stress (*P* < 0.01). Body weights in rats were significantly increased after treating with XYS and fluoxetine (Figure 1(b)).

### 3.2. XYS Effect on 1% Sucrose Preference in Rats with CIS

In order to know depressive behaviors in rats during immobilization stress period, 1% sucrose preference in rats was recorded on days 7th, 14th, and 21st. A repeated measure ANOVA showed significant differences among four groups (*P* < 0.01). Three times measurements of sucrose preference in each group also showed significant differences (*P* < 0.01).

There was no interaction impact on time points and groups. No statistical difference was shown in SFT in rats subjected to CIS for 7 days. After modeling for two and three weeks, sucrose preference rate in rats in model group was lower than those in control group (*P* < 0.01, *P* < 0.05, resp.); compared with the model group, XYS treatment group significantly increased the sucrose preference (*P* < 0.01, *P* < 0.05, resp.). Fluoxetine treatment group also significantly increased the sucrose preference compared with the model group (*P* < 0.05) (Figure 1(c)).

### 3.3. XYS Effect on Serum NE Levels in Rats with CIS

**Figure 2:**
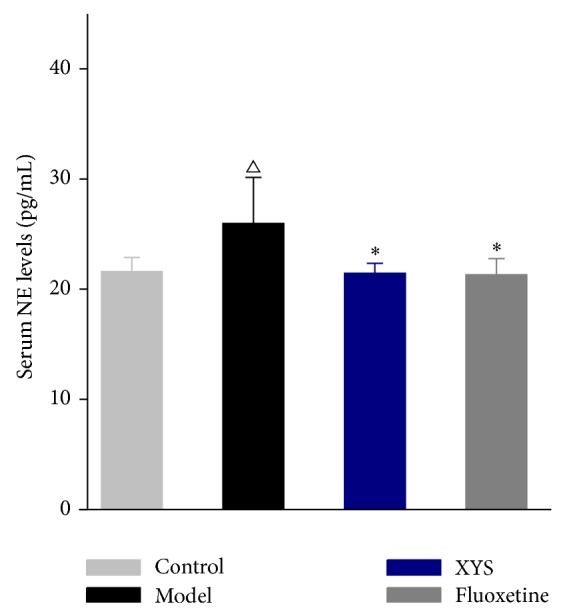
Effect of XYS on serum NE of rats with CIS. Data were expressed as mean ± SD, *n* = 12 per group. ^△^ *P* < 0.01 versus control, ^*^ *P* < 0.01 versus model.

In order to test whether chronic stress contributes to dysregulation of LC-NE system, peripheral NE concentrations were measured.  Figure 2 indicated that serum NE levels were significantly different among four groups (*P* < 0.01). The serum levels of NE were 21.59 ± 1.28, 25.97 ± 4.17, 21.43 ± 0.912, and 21.31 ± 1.46 (ng/mL) in the control, model, XYS, and fluoxetine treatment group, respectively. The statistics analysis showed that serum NE levels were significantly increased in the model group compared to the control group (*P* < 0.01). After XYS or fluoxetine treatment, NE levels were significantly decreased compared to the model group (*P* < 0.01).

### 3.4. LC Morphology and Effect of XYS on the Expressions of TH, DBH, and CRF in Rats with CIS

**Figure 3:**
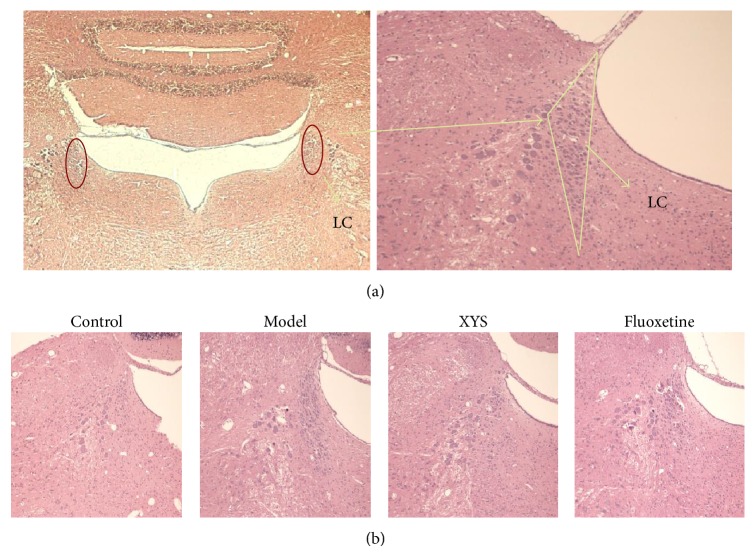
LC morphology in rats with CIS (H&E staining). (a) Morphology and site of LC in rat. (b) Morphology of LC in rats after modeling for 21 d CIS among four groups. *n* = 6 per group.

In order to evaluate the impact of CIS on gross LC morphology in rats, H&E staining was conducted in this study. The results showed that LC adjacent located to the fourth ventricle in the brainstem (Figure 3(a)). Furthermore, no discernible alterations were observed in LC structure in rats after exposure to CIS for 21 days (Figure 3(b)).

**Figure 4:**
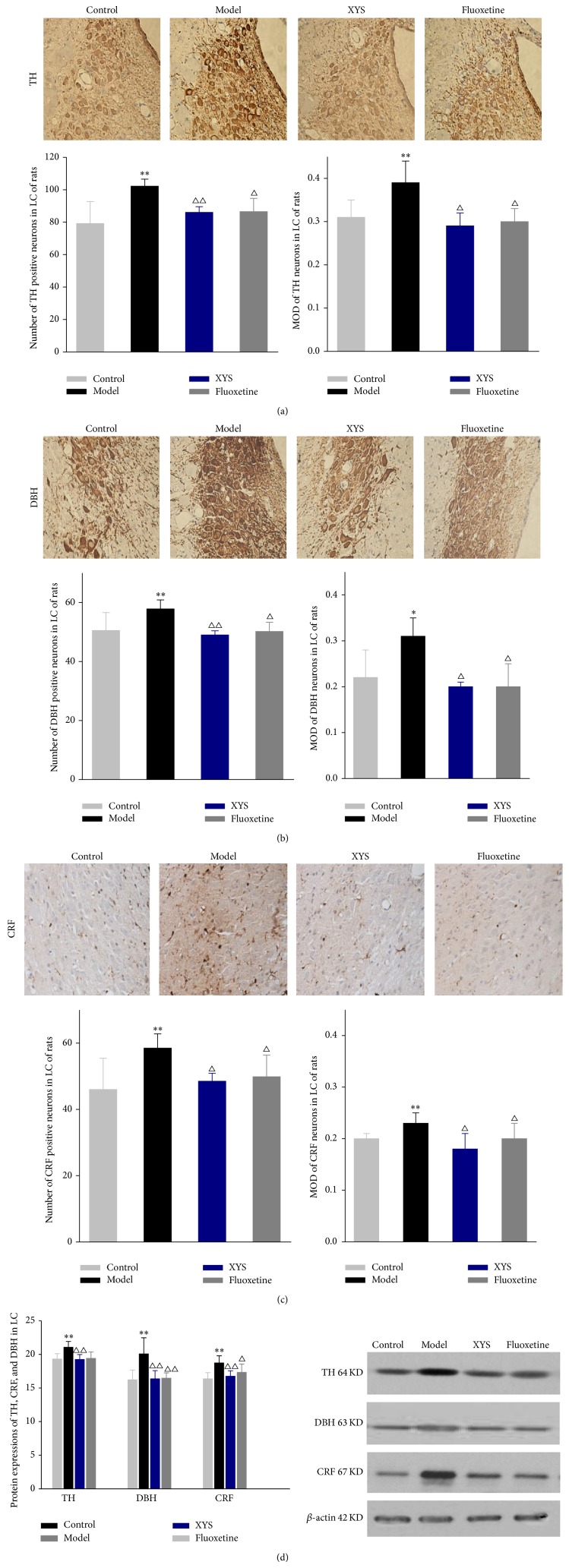
Effect of XYS on the expressions of TH, DBH, and CRF in LC of rats with CIS. ((a), (b), and (c)) The number of positive cells and mean density (SD) of TH, DBH, and CRF in LC of rats were determined by immunohistochemical staining. Data are expressed as mean ± SD, *n* = 6 per group. ^*^ *P* < 0.05, ^**^ *P* < 0.01 versus control, ^△^ *P* < 0.01 versus model. (d) The protein expressions of TH, DBH and CRF in LC of rats were measured by Western blotting. Data were expressed as mean ± SD, *n* = 6 per group. ^**^ *P* < 0.01 versus control, ^△^ *P* < 0.05, ^△△^ *P* < 0.01 versus model.

In order to determine whether CIS altered the function of LC neurons, TH, DBH, and CRF neurons were assessed by Immunohistochemistry. The results showed that LC expressed a number of TH, DBH, and CRF positive neurons in control group, 79.17 ± 13.56, 50.50 ± 6.09, and 46 ± 9.40, respectively. Quantity of the positive neurons of LC areas was increased and became dark brown, closely arranged, and round, shuttle or irregular shape of cell after modeling (Figures 4(a)–4(c)). In model group, the number of positive neurons of TH, DBH, and CRF was more than those of control group (*P* = 0.036, 0.003, and 0.002, resp.) and there was a significant difference. Similarly, MOD in model group was significantly enhanced compared to the control group (*P* = 0.001, 0.038, and 0.007, resp.). After treatment of XYS and fluoxetine, the number of positive neurons of TH, DBH, and CRF were significantly decreased compared with model group; likewise, MOD in these three neurons in model group was also significantly decreased compared to control group.

In order to examine protein expressions of TH, DBH, and CRF in rats were measured using Western blotting. Compared with the control group, protein levels of TH, CRF, and DBH in model group were significantly increased (*P* = 0.002, *P* < 0.01); protein levels of TH, CRF, and DBH in XYS and fluoxetine group were significantly decreased compared with the model group (*P* = 0.001, 0.003, *P* < 0.01). XYS and fluoxetine group had no obvious difference compared with the control group (*P* > 0.05, Figure 4(d)).

## 4. Discussion

Chronic immobilization stress is usually used as a model to assess the underlying alterations in cellular and molecular relevant to some depressive diseases [29–31]. Furthermore, antidepressant effect of XYS was approved by numerous literatures. Therefore, the present study used XYS to explore modulation of locus coeruleus-norepinephrine system in rats with CIS.

In order to adapt to various stress, a body can timely adjust physiological status. Moderate stress is beneficial to both body and mind, while excessive stress is disadvantage to a body's mental and physical health. This can be explained that rats adapted to CIS in the beginning in this study and were in an agitated state and prone to irritate and struggle after modeling for seven days. Rats could not adapt to this stress resulting in passive manifestations such as fatigue, sluggish, loose stool, body weight loss, and others after CIS for 14 days. This indicated that rats could not adjust themselves under excessive stress. After treating XYS that function is soothe liver and tonifying spleen, a trend to reverse the weight loss induced by stress was displayed.

Rodent is born with a preference for solutions or sweet foods. Decreased preference for sweet glucose solution in sucrose preference test means physical anhedonia, while therapies with chronic antidepressants can effectively reverse this reduction. SFT can detect the affective state or feelings of rodents [32]. To evaluate the depressive behaviors of rats with CIS in this experiment, a 1% sucrose preference protocol was used [27]; sucrose preference rate in first measurement was lower than those on the second and third. The reason is that rats may have an adaptation process in the sugar consumption test. There was a relatively stable trend on the second and third measurement, but they were lower than the first measurement. This showed that rats could not adjust themselves under excessive physical stress. XYS treatment was effective in the amelioration of anhedonia induced by CIS as well as positive control.

LC-NE system firing can lead to differential NE levels to release. Specifically, NE system not only can be activated in response to acute stress across many preclinical studies, but also can be elicited by chronic stress paradigms. Varied outcomes in experiment studies depend on characteristics of stressor or individual. Findings from chronic psychosocial stress such as immobilization or cold restraint stressors have showed high NE reactivities and high plasma NE concentrations [33]. Thus, increased serum NE concentrations were observed in the model group. Chronic treatment with XYS caused a decrease in serum NE concentrations similar to that seen after treating fluoxetine. This result showed that XYS may ameliorate the depressive-like behaviors in rats with CIS through regulating NE concentrations.

Both DBH and TH were used to reveal the norepinephrine expression not only in locus coeruleus neurons, but also in the NE axons or fibers across the entire neuraxis [34, 35]. Specifically, on the one hand, TH, the first enzyme in the norepinephrine biosynthesis, is widely utilized to identify the NE neurons [36]. On the other hand, DBH, the final enzyme in norepinephrine biosynthesis, is commonly introduced to use a specific biomarker for NE fibers. CRF contains axon terminals and CRF binding sites; diverse behavioral effects elicited by intracerebral CRF support that it can serve as a modulator in neuronal activity to mediate behavioral or physiological effects in rodents [37, 38]. Some substantial evidences also support that CRF has a neurotransmitter role in the locus coeruleus and moreover stated that CRF can be released into the locus coeruleus after locus coeruleus-norepinephrine system had been activated by various stressors and regulate locus coeruleus activation to facilitate behavioral or cognitive aspects of stress responses [39]. Therefore, in the present study, we analyzed the impact of immobilization stress on the expression of endogenous CRF and two genes involved in the biosynthesis of norepinephrine such as TH and DBH.

In this study, no discernible changes in LC structure were found by H&E staining, suggesting that rats exposed to CIS for 21 days retained their brain structural integrity. Our results suggested increased positive neurons and MOD in CRF and synthesis enzyme of NE such as TH and DBH in the brain catecholaminergic system in response to 21-day chronic stress. It may contribute to a coordinated upregulation of these three genes expression. But these upregulation levels were decreased in response to XYS treatment as well as fluoxetine. We further confirmed these results in protein level by Western blotting in this experiment, and mRNA level had been confirmed in the former research [40]. Interestingly, our previous studies showed mRNA levels of TH and DBH were increased in response to CIS for 1 h. These expressions were increased to the highest one after CIS for 3 h and then decreased after CIS for 6 h [25].

Taking these together, our studies indicate that LC-NE system can be activated by acute and chronic stress. But the activity of LC-NE system is rapid, transient in response to an acute stress. Contrarily, this activity is persistent in response to a chronic immobilization stress. We will further explore the mechanism of activation of the LC-NE system in response to chronic stress at different time points such as 7 d stress, 14 d stress, and other and identify differentially expressed proteins using a proteomics approach.

## 5. Conclusion

After exposure to an immobilization chronic stress, LC-NE system of rats can be activated resulting in a significant depression. Xiao Yao San can effectively improve depressive-like behaviors in rats. Further research is encouraged to detect the mechanism of activation of the LC-NE system in response to chronic stress in various time points and identify differentially expressed proteins or genes using an omics approach.
